# The Application of Computer Intelligence in the Cyber-Physical Business System Integration in Network Security

**DOI:** 10.1155/2022/5490779

**Published:** 2022-09-26

**Authors:** Shi Lin, Ma Yang, Yan Lu, Liquan Chen

**Affiliations:** ^1^School of Cyber Science and Engineering, Southeast University, Nanjing 210096, Jiangsu, China; ^2^College of Economics and Management, Nanjing University of Aeronautics and Astronautics, Nanjing 211106, Jiangsu, China; ^3^Jiangsu Financial Information Management Center, Nanjing 210024, Jiangsu, China

## Abstract

In order to address the false alarm detection problem caused by the inability to identify the transgression scene pages in the process of horizontal transgression detection, this study proposes a deep learning-based LSTM-AutoEncoder unsupervised prediction model. The model uses long short-term memory network to build AutoEncoder, extracts text features of page response data of horizontal transgression scenario, and reconstructs text features to restore. Meanwhile, it counts the error between the restored result and the original page response, judges whether the detection result of horizontal transgression is false alarm according to the error threshold of unknown page, and tests the effectiveness of the model effect under real business data by comparing it with other two algorithms, one-class SVM and AutoEncoder, which provides security for enterprise network business. The results show that the LSTM-AutoEncoder model achieves a more balanced index in terms of accuracy, precision, recall, and *F*1-score in the case of MAE, which is 0.3% more and 0.2% more than the case of MSE in terms of recall and accuracy. It is concluded that the LSTM-AutoEncoder model is more in line with the real business requirements, and the simple model architecture selected for this study can reduce the complexity of the model, speed up the prediction time of the model in the application phase, and improve the performance of the detection software. This indicates that this study has some application prospects in network security.

## 1. Introduction

Web security issues are closely related to our lives, among which transgression vulnerabilities are the most common. Transgression vulnerability is one of the business logic vulnerabilities, which is very common in current web applications [1, 2]. Horizontal override is one of the override loopholes, which means that users with the same authority can query, modify, delete, and add other people's information by modifying parameter variables to achieve illegal behavior [3, 4]. When a hacker exploits an override vulnerability, it is difficult to be monitored and processed by defense software because the attack behavior is no difference from normal user requests and does not contain sensitive characters and special characters [5]. In recent years, with the penetration of information technology into all aspects of people's lives, information security has begun to be frequently mentioned and concerned, especially in the modern society with highly developed computer technology and network technology. The development of all walks of life is closely related to information security, so issues involved in it must be paid enough attention to [6]. The problem of leaks caused by computer network security issues has also given people a warning. Network security problems can easily lead to personal information leakage and damage to reputation. To avoid such problems, people have put forward higher requirements for the use of computers and began to pay close attention to computer use and network security, so as to ensure the function of computer services [7]. Therefore, it is virtually important to detect the horizontal override vulnerabilities and repair the defects in time to avoid losses.

A lot of research work has been carried out [8–12]. In the actual business production process of an enterprise, it is not only necessary to enhance the security development awareness and code specifications of code developers, but also necessary to have good security detection for the upcoming web applications. For the detection of privilege escalation vulnerabilities, there are the following related studies. Sun et al. proposed a method of using static inspection to detect logical vulnerabilities in web applications and constructed sitemaps of visitors with different privileges through source code to determine whether there is unauthorized behavior [13]. Monshizadeh et al. proposed the MACE tool to detect privilege escalation vulnerabilities in large code bases, which found many serious and unknown vulnerabilities and achieved good results. Deepa et al. adopted a black-box method to identify logic flaws and vulnerabilities and constructed finite state machines by modeling expected behaviors [14]. Song et al. obtained the accessible links of the target website by means of URL link crawling, used different user permissions to access the linked pages, and parsed the web page responses to determine whether there are horizontal and vertical unauthorized vulnerabilities [15]. Ma et al. proposed a new permission control vulnerability detection method, that is, by establishing a five-layer model combined with authorization context information to detect whether the target system has permission control vulnerabilities [16]. Although there are many methods for unauthorized detection, the following problems exist in real business scenarios. First, the method of source code auditing is extremely expensive and requires relatively strong security knowledge personnel to spend a lot of time on detection. At the same time, due to the short iteration cycle and the huge amount of product code, it is difficult for security experts to take into account all the vulnerability points in web applications [17]. Secondly, the current automation level over-authority detection tools and methods often have the problem of false positives, which increases the cost of the verification of the detection results [18]. Although there have been many combined applications of artificial intelligence and network security [19–21], and the recognition of horizontal unauthorized scene pages has been realized, it is prone to the problem of false positives.

To address this problem, this study proposes a horizontal transgression detection model based on LSTM-AutoEncoder, which applies deep learning transgression scenario page recognition to transgression detection. And at the same time, this paper well solves the problem of false positives of horizontal transgression detection, ensures timely detection and repair of horizontal transgression vulnerabilities, as well as significantly improves the accuracy of horizontal transgression detection and reduces the work cost of manual review.

## 2. Horizontal Override Detection Based on LSTM-AutoEncoder

### 2.1. LSTM

**Figure 1:**
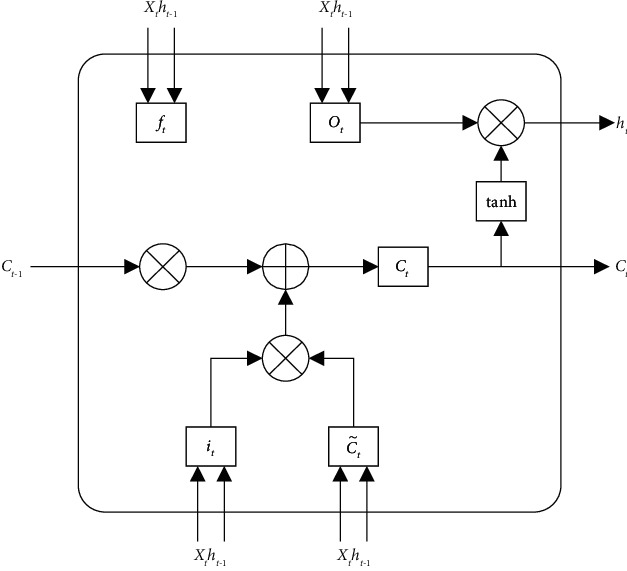
LSTM structure diagram.

The long short-term memory (LSTM) network was proposed by Hochreiter et al. [22, 23], using the control gate mechanism. The LSTM structure is shown in Figure 1, which is composed of multiple units. At present, the long short-term memory network has been applied in many fields. When this algorithm is used to process data, the data are generally divided into training values and observed values, the training values are used for network training, and the observed values are compared with the predicted values. For data with simple variation laws, this method has higher prediction accuracy [24].

In the LSTM structure diagram, *h*_*t*_ is the state output of the network at time *t*, and *X*_*t*_ is the input vector at time *t*. The state flow formula of the LSTM model is as follows.

The first is the forgetting stage, which will select historical information to be forgotten, *f*_*t*_ is the output state of the forget gate at time *t*, *σ* is the logistic sigmoid function, *W*_*f*_ is the weight matrix, and *b*_*c*_ is the bias vector:

(1) $$\begin{array}{c} f_{t}=\delta \left(W_{f}\cdot \left(X_{t},h_{t-1}\right)+b_{c}\right)f. \end{array}$$

Then, the state of the input gate is updated, which mainly includes the relevant factors of unauthorized identification. *i*_*t*_ is the output state of the input gate at time *t*, *b*_*i*_ is the bias vector, ${\tilde{C}}_{t}$ is a new candidate value vector created by tanh, *W*_*c*_ is the weight matrix, and *C*_*t*_ is the memory cell state at time t:

(2) $$\begin{array}{c} i_{t}=\delta \left(W_{i}\cdot \left(X_{t},h_{t-1}\right)+b_{i}\right). \end{array}$$

Candidate value vector formula:

(3) $$\begin{array}{c} {\tilde{C}}_{t}=\tanh\left(W_{c}\cdot \left(X_{t},h_{t-1}\right)+b_{c}\right). \end{array}$$

Memory cell state formula:

(4) $$\begin{array}{c} C_{t}=i_{t}\cdot {\tilde{C}}_{t}+f_{t}\cdot C_{t-1}. \end{array}$$

Finally, the result of the current state is output by the output gate, *o*_*t*_ is the output state of the output gate, *W*_*o*_ and *U*_*o*_ are the weight matrix, and *b*_*o*_ is the bias vector:

(5) $$\begin{array}{c} o_{t}=\sigma \left(W_{o}X_{t}+U_{o}h_{t-1}+b_{o}\right),\\ h_{t}=O_{t}\cdot \;\tanh\left(C_{t}\right). \end{array}$$

When LSTM's ability is adopted to process long-order data, training and processing the response data of the unauthorized scene web page can well ensure the contextual validity of the unauthorized scene data.

### 2.2. LSTM-AutoEncoder

AutoEncoder usually consists of an encoder and a decoder [25]. Hinton et al. proposed a prototype of auto-encoding to test their Boltzmann machine learning algorithm [26]. First, the web page response data *x* is input. Then, the encoding stage encodes the input response data to the hidden layer *h* through the mapping function *f* : *h*=*f*(*x*), and the decoding stage passes the mapping function *g* : *x*′=*g* in the decoding stage (*h* decodes the *h* of the hidden layer into new web page response data). The reconstruction error of the encoder is

(6) $$\begin{array}{c} \min{\left\|x-g\left(f\left(x\right)\right)\right\|}_{2}^{2}. \end{array}$$

By trying to use the AutoEncoder to convert the web page response data into text vector into a new response text vector, and by comparing the difference between the original text vector and the newly generated text vector, the category of the web page response data is judged.

LSTM-AutoEncoder was first proposed by Srivastava et al. to learn representations for video sequences. Currently, LSTM-AutoEncoder is widely used for extreme temporal prediction and anomaly detection in text sequences [27, 28]. The web page response of the horizontal unauthorized scene page is a text sequence, in which the unauthorized related scene page can be considered as a normal page, and the non-unauthorized related page can be considered as an abnormal page. Modeling based on this design idea can realize the detection of unauthorized scene pages.

The evaluation indicators of the LSTM-AutoEncoder model are mean squared error (MSE), mean absolute error (MAE), and commonly used evaluation indicators in machine learning, which contain precision, accuracy, recall, and *F*1-score [29]. The calculation formulas of MAE and MSE are as follows, where *X*_prediction_ is the predicted value each time, *X*_real_ is the actual value, *N* is the total number of times, and *t*, *i* is the current number of times:

(7) $$\begin{array}{c} \text{MSE}=\frac{1}{N}\sum_{t=1}^{N}{\left(X_{\text{prediction,}t}-X_{\text{real},t}\right)}^{2}, \end{array}$$

(8) $$\begin{array}{c} \mathrm{MAE}=\frac{1}{N}\sum_{i=1}^{N}\left(X_{\text{prediction,}i}-X_{\text{real,},i}\right). \end{array}$$

The calculation formulas of precision, accuracy, recall, and *F*1-score are as follows, where TP is a true positive example, FP is a false positive example, TN is a true negative example, and FN is a false negative example:

(9) $$\begin{array}{c} \text{precision}=\frac{\text{TP}}{\text{TP}+\text{FP}},\\ \text{recall}=\frac{\text{TP}}{\text{TP}+\text{FN}}. \end{array}$$

The formula for precision and *F*1-score is as follows:

(10) $$\begin{array}{c} \text{accuracy}=\frac{\text{TP}+\text{TN}}{\text{TP}+\text{TN}+\text{FP}+\text{FN}},\\ F1-\text{score}=2\cdot \frac{\text{precision}\cdot \text{recall}}{\text{precision}+\text{recall}}. \end{array}$$

The input of the LSTM-AutoEncoder model is the vectorized horizontal unauthorized scene page text, and the tokenizer module of the Keras framework is used for text preprocessing. First, the tokenizer instance object is built and passed in the stop character through the filters parameter, in which the maximum reserved phrase is set to 20, 000. Then, the training data for fitting are used to obtain a dataset text dictionary that can convert text into sequences. When new data need to be predicted, this dictionary can be used for conversion. Finally, the converted text sequence is deformed into a three-dimensional array required by the input of the LSTM layer, which is used as the training data to input the model.

Table 1 shows the architecture code of LSTM-AutoEncoder. The encoder and decoder are composed of two LSTM units, respectively. The RepeatVector layer is used to change the data dimension, and the TimeDistributed layer and the dense layer are used to deal with the many-to-many situation of the data decoding dimension.

### 2.3. Horizontal Override Detection Based on LSTM-AutoEncoder

In web applications, horizontal override occurs among users with the same privileges [30]. Its main feature is that users with the same authority can forge other users' behaviors by modifying parameters under the user's authority, and can view and use other users' data. The flowchart of LSTM-AutoEncoder-based horizontal override detection among users with the same privileges is as follows.

**Figure 2:**
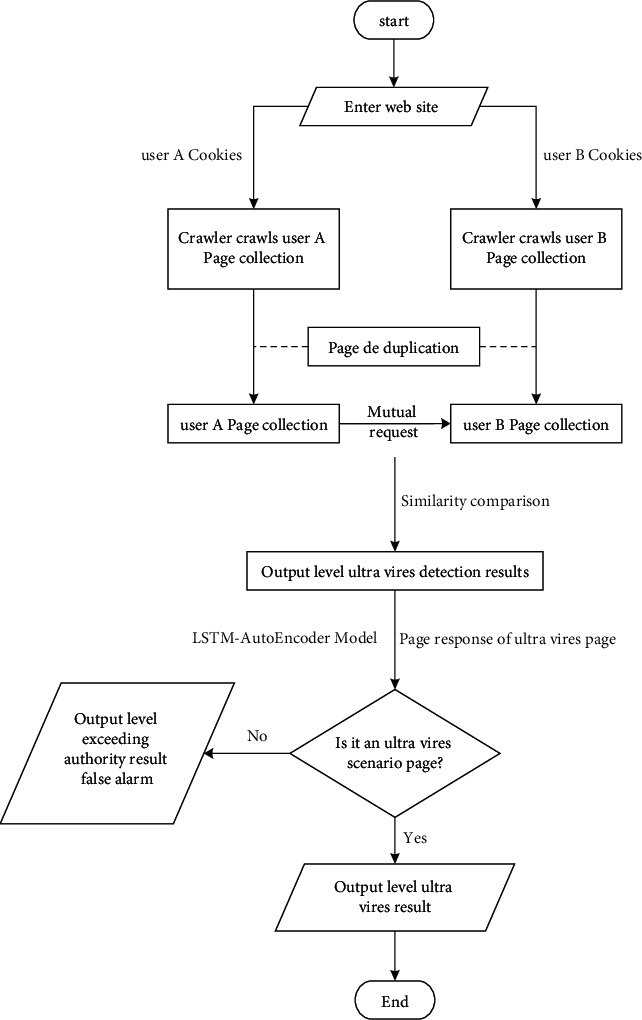
Flowchart of horizontal override detection based on LSTM-AutoEncoder.

As can be seen from Figure 2, the detailed description of the horizontal unauthorized detection process based on LSTM-AutoEncoder is as follows.

The first step is to use user A's cookie and user B's cookie to visit the target website, respectively, use crawler technology to automatically crawl target page links and web page responses by carrying user cookies, and build a set of response pages for users A and B.

The second step is to deduplicate the pages of users A and B according to URL links and page responses. Page deduplication mainly removes the pages that both users A and B can access under the same permissions as the static pages of the web pages that are being accessed and the shared pages of the web pages. By comparing the URL links, request body similarity and web page similarity of users A and B, the pages in which the URL link is the same and the page similarity and request body similarity are 100% are identified as duplicate pages. Through the above method, the set of deduplicated pages of users A and B is obtained.

The third step is to use the deduplication page sets of users A and B to request each other. Concretely, the cookie of user A is used to request the URL link in the page collection of user B, and at the same time the parameters in the URL are replaced with the request parameters of user B, so as to get the page response of user A requesting user B. Then, the similarity between the page response of user A and the page response of user B is compared under the same link. When the similarity is greater than the set threshold (in the actual test process, the threshold is set to 98.4% according to the results of multiple tests), it is considered that this link is unauthorized. Similarly, user B's cookie is used to request user A's URL link to obtain another set of unauthorized links. The link set after deduplication of the two sets of links is the link set that is preliminarily judged to have an unauthorized vulnerability.

In the process of automatic level override detection, there are often a lot of noise data (such as UUID, timestamp, and other data dynamically generated by JS) in web pages. The degree is less than 100%, has not been deduplicated, and enables in the third step of the mutual request detection; the page similarity of the response page after the request of users A and B is higher than the threshold, thereby resulting in a false positive for the horizontal unauthorized detection result. The horizontal unauthorized vulnerability only occurs in the horizontal unauthorized scene page, so the problem of horizontal unauthorized false positive can be solved by constructing a deep learning model to detect whether the result page is an unauthorized scene page.

The fourth step is to perform data preprocessing on the response page data in the horizontal unauthorized detection result and then perform the unauthorized scene page detection through the LSTM-AutoEncoder model. When the model determines that the page type is an unauthorized scene page, it is concluded that the page has a horizontal unauthorized vulnerability. Otherwise, it is considered that the horizontal unauthorized link determined in the third step does not belong to the horizontal unauthorized scene page, and there is a false positive.

## 3. Test Analysis

### 3.1. Experimental Data and Experimental Environment

LSTM-AutoEncoder belongs to the unsupervised single-classification model, so only the unauthorized scene pages need to be collected during the model training stage. The number of pages without business data interaction in the web application website is much larger than the number of pages with user business data interaction. Therefore, in the model testing stage, the non-unauthorized scene pages used to test the performance of the model are randomly selected from the website pages without business interaction in the training set website. In order to fit the real business scenario, the experimental data collected in this paper come from a large number of web application websites such as Internet forums and e-commerce, which involves pages of unauthorized scenarios such as personal information, order payment, inquiry, invoice, complaint, and suggestion. Furthermore, the corresponding type of website directory is found through the e-commerce index website and forum index website and the links are crawled. Then, the website is registered and by the selenium automated testing tool, the script set is used to obtain the web page response of the unauthorized scene page rendered by the browser after registration. Meanwhile, the identification and labeling of the unauthorized scene page are carried out by the security service personnel, the data collection process consumes a total of 60 man-days, and Jieba is used to segment the collected web page responses. Considering that in existing mainstream web development frameworks such as React, Vue, and other web page source codes dynamically generated by JavaScript, English character tags are often randomly generated and contain very few data features, English data are discarded during word segmentation, and stop words are removed according to the Chinese stop words table and the place name information table. After cleaning, a total of 12,000 unauthorized scene page data for model training and 1,200 nonauthorized scene page data for testing model performance are collected. The data splitting function train_test_split in the scikit-learn framework is used to divide the data into training set, test set, and validation set according to the ratio of 0.8:0.1:0.1, and the sequence length of each sample data is unified to 1000 through the pad_sequence function of the Keras module. The dataset statistics are shown in Table 2.

Because theresponse text of the web page is too long, Table 3 lists the first 15 texts of some dataset examples in the training set.

**Figure 3:**
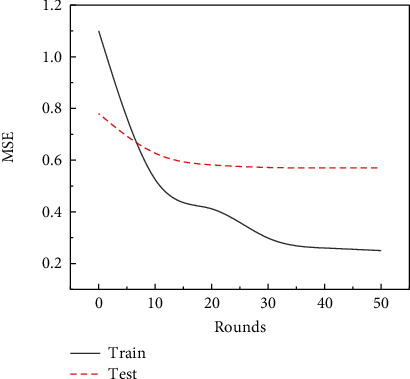
Model MSE loss drop chart.

The experimentalprocess is the same as the detection flowchart given in Figure 3. Firstly, theautomatic horizontal unauthorized detection is used to perform horizontal unauthorized detection on the target website, and then the web page response of the target link corresponding to the detection result judged to be unauthorized is input into the LSTM-AutoEncoder model to detect the unauthorized scene page. If the output of the LSTM-AutoEncoder model is yes, the web page corresponding to the link is an unauthorized scene page, and it is determined that the link exists as a horizontal unauthorized scene, whereas if the detection result is no, it means that the link is not an unauthorized scene page, and the unauthorized result is a false positive. The experimental environment is as follows: LSTM-AutoEncoder model uses Python 3.6 as the development language, Keras == 2.1.2 as the high-level packaging framework, and tensorflow-gpu == 1.10.0 as the deep learning framework, and the CPU is Intel i7-7700K, GPU model training for NVIDIA 2080 Ti Windows machines.

### 3.2. Experimental Method

The following methods are used for the judgment of how to use the LSTM-AutoEncoder model for horizontal unauthorized scene pages.

**Figure 4:**
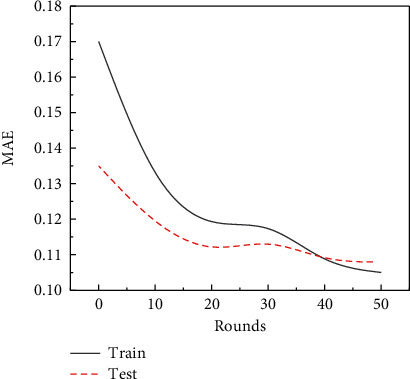
Model MAE drop graph.

First, model training is performed using MSE and MAE as loss functions, respectively. After continuously adjusting the unit size, batch size, activation function, and round epoch, the optimal model with units = 64, batch size = 32, epoch = 50, and activation function of ReLU is obtained. Figures 3 and 4 show, respectively, MSE and MAE LOSS descent plots of the best model.

It can be seen from Figure 3 that the MSE of the training value is significantly higher than the test value at the first 10 training times, and then, with the increase of training round, the MSE of the training value is significantly lower than that of the test value and finally remains around 0.2, which shows that with the progress of the experiment, the MSE error of the test is gradually reduced, and the test prediction results are more and more accurate. In addition, Figure 4 shows the decrease of the model's mean absolute error loss. In this study, with the increase of training times, the MAE of both the training value and the test value shows a downward trend, and after 40 times of training, the MAE of the test value is higher than that of the training value, which indicates that when the number of training times reaches a certain number, the accuracy will reach the best accuracy, and the data prediction is most feasible at this time.

**Figure 5:**
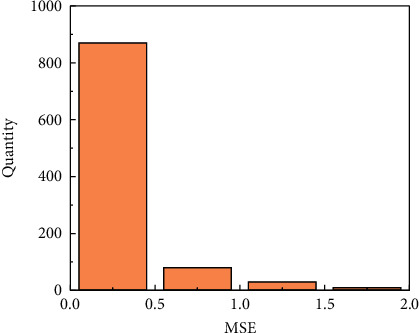
Distribution of MSE.

**Figure 6:**
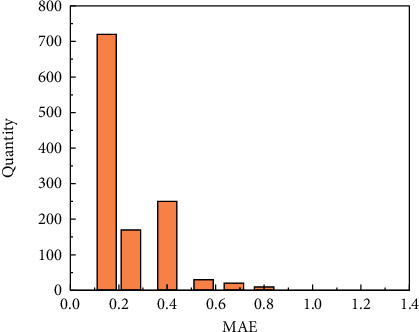
Distribution of MAE.

Secondly, the best models of MSE and MAE are adopted to predict the original training set, respectively, and the calculation formulas of MSE and MAE (Equations (7) and (8)) are used to obtain the MSE and MAE distribution intervals between the predicted value and the original value. The distribution interval determines the threshold for the restoration error. The distribution interval of MSE and MAE is shown in Figures 5 and 6, the threshold of MSE is 1.75, and the threshold of MAE is 0.65. As can be seen from Figure 5, when the MSE error is within 0.5, the number proportion is the highest, which indicates that the prediction effect is better, whereas when the number of MSE is above 0.5, the number proportion is less, which indicates that the model has higher prediction accuracy. Compared with MSE, the distribution range of MAE is also mainly within 0.5, of which the number of data within 0.2 accounts for the highest proportion, followed by the part with MAE of 0.4. The data plot of MAE and MSE shows that the model has better prediction effect.

Finally, the MSE and MAE distributions of the validation set and negative samples are calculated, and according to the threshold, the TP, TN, FP, and FN values of the model in the case of MSE and MAE can be calculated. Assuming that the threshold is *k*, test is the test data, error is the error data, and sum is the sum of the data; the calculation method is as follows:

(11) $$\begin{array}{c} TP=\text{sum}\left(\text{test}<k\right),\\ TN=\text{sum}\left(\text{error}>k\right),\\ FP=\text{sum}\left(\text{error}<k\right),\\ FN=\text{sum}\left(\text{test}>k\right). \end{array}$$

**Figure 7:**
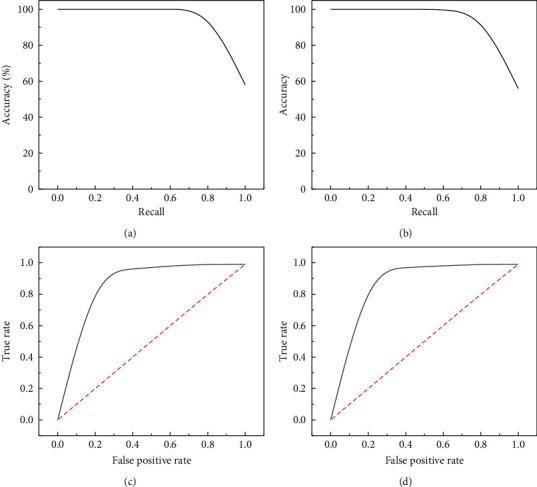
Accuracy and true rate graph, (a) MSE precision-recall plot; (b) MAE precision-recall plot; (c) MAE subject characteristic curve; (d) MSE subject characteristic curve.

The ROC curve is usually used to evaluate the quality of a classifier. The abscissa is TPR, and the ordinate is FPR, which, respectively, represent the probability of the classifier classifying positive examples into pairs and the probability of the classifier wrongly classifying negative examples. Moreover, AUC represents the area under the ROC curve, and the closer the AUC is to 100%, the higher the predicted value is. The precision curve of the image and the ROC curve can be obtained from the TP, TN, FP, and FN values, as shown in Figure 7.

In order to verify the effectiveness of this paper, the one-class SVM model and the AutoEncoder model are trained and compared with the same data preprocessing method and the same training data. Among them, the one-class SVM model uses the preset model and default parameters in the scikit-learn module for training, the AutoEncoder model uses a double-layer fully connected layer as the encoder and decoder, and the unit parameter is 32 for training. The comparison results of the three models for the validation set are shown in Tables 4to 6.

According to the experimental results, in the one-class model, the precision of the model reaches up to 0.974, indicating that the one-class model has a high recognition rate for unauthorized scene pages in the dataset. However, the recall rate of the model is only 0.473, indicating that for many non-unauthorized scene web pages, the model identifies them as unauthorized scene pages. Therefore, in the actual business process, the one-class SVM model cannot solve the problem of false positives of horizontal overreach.

The recall rates of the AutoEncoder model in the case of MAE and MSE are 0.988 and 0.994, indicating that the case of the correct horizontal unauthorized scene page occupies a high proportion to all the horizontal unauthorized samples, but the average precision rate of such model in the case of MAE and MSE is lower by about 3% compared to that of LSTM-AutoEncoder indicating that the proportion of pages predicted by the model as horizontal unauthorized scenes is only 0.89%. Therefore, the overall performance of the AutoEncoder model is weaker than the LSTM-AutoEncoder model. The *F*1-score often represents the harmonic average of the precision rate and the recall rate, which is consistent with the business requirements for page detection in horizontal override scenarios. It can be seen from Table 3 that the accuracy, precision, recall, and *F*1-score of the LSTM-AutoEncoder model in the case of MAE have reached a relatively balanced index. Compared with the case of MSE, the *F*1-score of MAE is 0.3% more, and the accuracy rate of MAE is increased by 0.2%, which is more in line with real business needs. Therefore, the LSTM-AutoEncoder model trained with MAE as the loss function is used as the detection model of the online environment to realize the detection of horizontal unauthorized scene pages.

According to the characteristics of the LSTM network, its feature extraction ability for the contextual web page response text sequence is stronger, which makes the comprehensive performance of the model more excellent. In addition, the multi-layer LSTM codec has been tried in the experiment, but the accuracy of the model is only increased by 0.001%, and even the accuracy of the four-layer network has dropped. The recognition of scene pages brings better results. In the actual business inspection scenario, the efficiency of the model is the key to the rapid operation of the detection system. Blindly pursuing the accuracy rate and abandoning the performance parameters must be avoided in the development process. After the time-consuming test of the prediction module, the prediction time of the model is 0.04 s, which meets the business needs of calling a large number of model predictions. After weighing the pros and cons, choosing the simple model architecture as shown in Figure 3 can reduce the complexity of the model, accelerate the prediction time of the model in the application stage, and improve the performance of the detection software.

## 4. Conclusion

Horizontal transgression vulnerability is particularly important to the business data security of companies and users. In this paper, we use the sample data of web application transgression scenarios and train the LSTM-AutoEncoder model to identify the pages of transgression scenarios, which solves the problem of false positives in traditional transgression detection and improves the accuracy of transgression detection. The experimental data show that the LSTM-AutoEncoder model has certain accuracy advantages over the traditional one-class SVM model and AutoEncoder model under certain amount of data scale and also has great advantages in processing web text sequences with contextual relationships, which is as follows [31]:

1. In the one-class model, the model reaches a very high precision of 0.974 and has a high recognition rate for unauthorized scene pages in the dataset. However, the recall rate of the model is only 0.473, which makes the non-unauthorized scene web page be recognized by the model as an unauthorized scene page.
2. The recall rates of the AutoEncoder model in the case of MAE and MSE are 0.988 and 0.994, respectively, and the cases that are correctly judged as horizontal unauthorized scene pages occupy a high proportion to all the horizontal unauthorized samples. However, the average precision rate of such model in the case of MAE and MSE is lower by about 3% compared to that of LSTM-AutoEncoder.
3. In the case of MAE, the precision, accuracy, recall, and *F*1-score of the LSTM-AutoEncoder model have reached a relatively balanced index. Compared with the case of MSE, its *F*1-score is 0.3% higher and the accuracy rate is 0.2% higher, which is more in line with real business needs.
4. After the time-consuming test of the prediction module, the prediction time of the model is 0.04 s, which meets the business needs of calling a large number of model predictions. After weighing the pros and cons, choosing the simple model architecture as shown in Figure 3 can reduce the complexity of the model, accelerate the prediction time of the model in the application stage, and improve the performance of the detection software.

The application of AI and network security is at a relatively early stage of development, and this paper provides a good application case. However, the experimental data in this paper also have certain shortcomings. In future research, it is necessary to extend the collection of training data to more types of pages of transgression scenarios in a large number of web applications on the Internet, so as to improve the generalization ability of the model and make it possible to test more transgression scenarios.

## Figures and Tables

**Table 1:** LSTM-AutoEncoder code architecture diagram.

Inputs = input (shape = (train_data.shape [1]), train_data.shape [2])
# ENCODER
*L*1 = LSTM (64, activations = relu) (inputs)
*L*2 = RepeatVector (train_data.shape [1]) (L1)
# DECODER
*L*3 = LSTM (64, activations = relu, return_sequences = True) (L2)
Outputs = TimeDistributed (dense (train_data.shape [2])) (L3)
LSTM-AE = Model (inputs = inputs, outputs = outputs)
Inputs = input (shape= (train_data.shape [1]), train_data.shape [2])
# ENCODER
*L*1 = LSTM (64, activations = relu) (inputs)

**Table 2:** Dataset statistics.

Data type (number)	Training set	Test set	Validation set	Statistics
Unauthorized scenario page	9600	1200	1200	12000
Nonurban scenario page	0	0	1200	1200

**Table 3:** Examples of training set datasets.

Number	Example
1	Connect the poster QR code to log in using the Weibo account to access the poster account password verification code and change in dynamic prompt guarantee must
2	Screenshot of the client's wonderful download, watch video, homepage channel, special event reminder, homepage account setting recommendation
3	Car rental global-car rental home car store activities car rental-login registration-hello-order assets-account logout
4	Enter the mobile phone number-retrieve the user name-home member login service-retrieve the user name-mobile phone number verification code-now available
5	Free registration, complete information, and login problems
6	The user logs in to the homepage of Super Comics-update ranking-search and read-clear record-login synchronization-read click
7	Account-personal center-home interactive-home topics-Q&A center-index market-market data-announcement home
8	Merchant center, return to the homepage, welcome to the settings menu, release open store settings, profile account name, contact

**Table 4:** One-class SVM prediction results.

Precision	Accuracy	Recall	*F*1-score
0.974	0.730	0.473	0.636

**Table 5:** AutoEncoder prediction results.

Loss function	Precision	Accuracy	Recall	*F*1-score
MSE	0.895	0.940	0.994	0.942
MAE	0.897	0.940	0.988	0.940

**Table 6:** LSTM-AutoEncoder prediction results.

Loss function	Precision	Accuracy	Recall	*F*1-score
MSE	0.931	0.942	0.955	0.942
MAE	0.920	0.944	0.971	0.945

## Data Availability

The dataset can be accessed upon request to the corresponding author.
